# Therapists' time allocation in guided digital mental health interventions – A scoping review and case study

**DOI:** 10.1016/j.invent.2026.100983

**Published:** 2026-07-30

**Authors:** Iris C. Brunner, Linn N. Støme, Robin M.F. Kenter

**Affiliations:** a ForHelse Research centre for digital mental health, Haukeland University Hospital, Haukelandsbakken 2, 5009, Bergen, Norway; b Department of Research and Innovation, Oslo University Hospital Division of Mental Health and Addiction, Oslo, Norway

**Keywords:** Mental health, Guided digital interventions, Time, Implementation

## Abstract

**Background:**

Digital Mental Health Interventions (DMHIs) that include therapist support achieve higher adherence and greater effectiveness than fully self-help applications. Compared to face-to-face therapy, guided DMHIs can expand access by reducing the amount of therapist time required while still supporting a therapeutic relationship. However, “therapist time” is operationalised inconsistently. Many studies appear to report only direct patient contact (e.g., messaging, calls) or platform-based monitoring, leaving uncertainty about whether other patient-related tasks, such as documentation, are included.

This scoping review examined how much therapist or other health-care professional (HCP) time is reported when delivering guided DMHIs or stepped/blended care interventions with a core digital component. A secondary aim was to describe the categories of time allocated. A case study from the Norwegian eTreatment service complemented the review.

**Methods:**

Following PRISMA-ScR guidelines, we searched for studies that quantitatively reported therapist or HCP time in guided or blended DMHIs. Eligible studies were in English, used a core digital treatment component with human support, and targeted mental health conditions. Pure self-help apps and teletherapy-only interventions were excluded.

**Results:**

Fifty-four studies published between 2005 and 2026 were included. Therapists spent a mean of 214.57 min (3 h 35 m; SD 214.76; range 46–1198; median 146; IQR 142.98) per patient per intervention, with an average program duration of 10.6 weeks. Forty-eight studies quantified only direct patient-related work such as calls, messaging, or platform monitoring. Other time categories were rarely quantified. In the case study, 73.7% of patient-related time occurred outside the platform, including diagnostic interviews.

**Conclusions:**

Studies predominantly report direct contact and monitoring, likely underestimating actual time use. Still, guided DMHIs appear to offer meaningful time savings compared to face-to-face therapy. More comprehensive reporting of direct, indirect, and administrative time would better support scaling and implementation.

## Highlights

• Therapist time in guided digital mental health interventions (DMHIs) is often underreported.
• Existing reports mainly capture direct interactions within the digital platform.
• Despite this, DMHIs still offer substantial therapist time savings.
• Comprehensive assessment of all time components is beneficial for successful implementation.

## 1 Introduction

A substantial body of research shows that Digital Mental Health Interventions (DMHIs), especially internet-delivered cognitive behavioural therapy (ICBT), can be as effective as face-to-face treatments for common disorders such as depression and anxiety (Titov et al., 2017; Etzelmueller et al., 2020; Hedman-Lagerlöf et al., 2023). They are increasingly promoted to address global shortages in mental health services (Patel et al., 2018; Olthuis et al., 2016). DMHIs can expand access for remote communities (Weightman, 2020), support patients on waiting lists (Peipert et al., 2022), and complement other treatments (Nelson et al., 2024). Despite these advantages, implementation in routine care remains limited (Dülsen et al., 2020; Graham et al., 2020a; Appleton et al., 2023). Still, DMHIs represent a promising way to increase treatment access and meet the unmet needs of people with mental health disorders worldwide (Schueller and Torous, 2020).

While patient needs drive the expansion of DMHIs, workforce challenges also play a role. Healthcare personnel shortages are expected to worsen due to demographic shifts (Almeida-Meza et al., 2025), Enhancing working conditions through the use of DMHIs, including increased flexibility in working hours and opportunities for remote work, may help reduce stress and improve retention (Dülsen et al., 2020; Standal et al., 2026). From an environmental perspective, reducing travel through telehealth can significantly lower carbon emissions (Usher et al., 2024), as clearly demonstrated during the pandemic (Sharma et al., 2023).

Evidence suggests that DMHIs incorporating therapist support achieve higher adherence and effectiveness compared to fully self-help applications in ICBT studies (Vernmark et al., 2024; Karyotaki et al., 2021). While self-help applications require promotion, integration, and maintenance, therapist-guided DMHIs introduce additional complexity, including training, supervision, workflow integration, and ongoing support to ensure acceptance among clinicians and patients. Consequently, understanding the human resource requirements for guided DMHIs is critical, as these directly impact costs and staff availability.

Although guided DMHIs involve therapist or other health care professional (HCP) time, a profound reduction of therapist time compared to face-to-face therapy was found in multiple studies (Axelsson et al., 2020; El Alaoui et al., 2017; Clark et al., 2023a). For example, Axelsson et al. found that therapists spent 78% less time delivering internet-based CBT (ICBT) compared to face-to-face CBT for health anxiety (Axelsson et al., 2020). Similarly, El Alaoui et al. (2017) reported a mean use of effective psychologist time in ICBT of 189.60 (53.77) minutes versus 499.78 (30.91) minutes in a group CBT setting (El Alaoui et al., 2017). Wright et al. (2005) observed 50% reduction of therapist time when comparing face-to-face CBT to ICBT in a blended approach (Wright et al., 2005). Reviews by Esfandiari et al. (Esfandiari et al., 2021), and Vigerland et al. (Vigerland et al., 2016), cite reductions of up to 85%.

While these savings appear substantial, there are different operationalisations of “therapist time”. Many studies only account for direct patient contact, i.e. asynchronous and synchronous interaction (messaging, phone and video calls) or monitoring of progress on the platform (Clark et al., 2023a; Andrews et al., 2023). Other patient-related time, such as record-keeping or communication with other HCPs are neglected or only estimated (Clark et al., 2023a; Ritola et al., 2022). This inconsistency limits the ability of health services to accurately estimate workforce requirements for scaling DMHIs. A realistic estimate encompassing both direct patient-related time, and indirect patient-related time would provide valuable guidance for scaling and implementing DMHIs (Vigerland et al., 2016).

In Norway, guided internet delivered psychological treatment has been tested and progressively implemented within specialist mental health services since the late 1990s with nationwide rollout initiated in 2025. Scaling their use is a national health priority. Existing eTreatment units deliver online programs that address the most common mental health disorders such as panic disorder, social anxiety, and depression, among others.

In this scoping review, we examined how therapist time allocation is reported in the literature and used the findings to outline key categories of time use. In guided DMHIs, support may be provided by different professionals, including psychologists, therapists, nurses or other trained staff. In this paper, we use the terms therapists and HCPs to refer to all professionals providing guidance within DMHIs.

To complement the review, we conducted a case study in eTreatment Bergen to assess therapist time allocation and evaluate the categories of time allocation in practice.

The objective was to provide a realistic estimate of therapist time allocation in guided DMHIs to inform implementation.

Research questions

- How much therapist or other HCP time is reported for providing guided DMHIs?
- What categories of time allocation are reported?

## 2 Methods – scoping review

### 2.1 Design

This review followed the reporting guidelines described in the PRISMA extension for scoping reviews (https://www.prisma-statement.org/scoping, n.d.). The following databases were searched for original publications, reviews, and other potentially relevant literature: PubMed, PsycINFO, Embase, Cinahl, and Web of Science. We searched for any studies that reported the time allocation of psychotherapists or other health care professionals providing guided DMHHs. Psychological interventions delivered through or supported by digital technologies are described using numerous, sometimes overlapping terms, including *internet-based treatment*, *internet-delivered cognitive behavioural therapy* (ICBT/iCBT), *computerised CBT*, *eHealth*, *e-mental health*, and *blended care*. Experts have agreed that this terminological inconsistency has important consequences for literature searching, comparison across studies, and cumulative knowledge development, although a single shared terminology has not yet been established (Smoktunowicz et al., 2020). In this review, we use *digital mental health intervention* (DMHI) as a pragmatic umbrella term because the eligibility criteria encompass multiple therapeutic approaches, delivery formats, and digital technologies and are not restricted to CBT. This terminology does not imply that all eligible interventions are equivalent. The operational eligibility criteria defined the interventions included, and the search strategy incorporated multiple related terms to account for variation in terminology.

Only quantitative data reported in minutes and/or hours were taken into consideration. Overview papers where the implementation of specific interventions was described, and time allocation was reported were also included. We chose to focus on treatments that were designed as guided online interventions. We also included blended care approaches where digital components were intended to reduce face-to-face sessions. Likewise, studies where stepped care was applied by providing varying levels of support during an otherwise consistent online treatment, based on initial screening results, were included.

The protocol has been registered at Open Science Framework (OSF) registry and can be found at https://osf.io/5bzvx. Eligibility criteria are listed in Table 1.

### 2.2 Eligibility criteria

**Table 1:** Eligibility criteria.

Inclusion criteria	Exclusion criteria
- Any type of studies on evidence-based guided DMHIs that report therapists' time allocation - Published in English, peer-reviewed journals	- White papers, policy statements, and other grey literature - Studies published as a commentary, opinion piece, editorial or letter to the editor, conference abstract without full-text article, or study protocol.
- Primary intervention delivered through a digital platform, where the digital component constitutes a core mode of therapeutic delivery.	- Interventions consisting solely of synchronous teletherapy (e.g., video/phone calls)
- Aimed at treatment of mental health disorders (e.g., depression, anxiety, OCD, PTSD, eating disorders, psychosis) as a primary or secondary complaint	- Exclusively targeting somatic conditions
- Guided interventions that include human therapeutic support by a professional, paraprofessional, or trained peer	- Exclusively self-guided (unguided) DMHIs without human therapeutic support

### 2.3 Search strategies

Two academic librarians assisted with the search and helped formulating and testing the search strings. We had compiled a list of 10 core articles to validate and refine the search terms. However, since information on time allocation was often absent from titles and abstracts, we employed an extended manual search along with backward and forward snowballing. The above-mentioned databases were searched from first publications until present. Search strings included the following keywords: “digital mental health interventions”, “internet-delivered”, “therapist or clinician or psychologist”, “time or hour or minute”.

For complete search queries for the different data bases see Supplementary File 1.

### 2.4 Screening process

For the deduplication of distinct duplicates, the software ASYSD https://camarades.shinyapps.io/ASySD/ was used (Hair et al., 2023). Subsequently, citations were uploaded to Rayyan, a review software that allows for deduplication, blinded screening by several reviewers, and opportunities to add other relevant literature not included in the citations from the search strings applied (Ouzzani et al., 2016). Three reviewers conducted a first screening round independently on all included articles. Based on their titles and abstracts they were categorised as “Included”, “Excluded”, or “Maybe”. All types of reviews were categorised as “Maybe” for later inspection of potentially relevant studies.

After this initial screening round blinding was removed and the results compared. All cases of disagreement were discussed and resolved with the help of a fourth reviewer. In a second screening round the full texts were examined for information on time therapists time allocation and included if criteria were fulfilled.

As a result of the screening process eligibility criteria were adapted. Originally, only studies conducted in routine care were to be included. However, the first screening revealed that it could be difficult to differentiate routine care from academic settings. We found many examples of new treatments applied in established online clinics, e.g. (Gandy et al., 2020; Johnston et al., 2011), and/or compared to standard treatments, e.g. (Murphy et al., 2020; Spence et al., 2011). The divide between academic and routine care setting seemed unclear and difficult to ascertain. Consequently, we agreed on including all articles where time use in guided DMHIs was reported.

### 2.5 Data extraction

Two reviewers extracted data independently from all eligible studies. Extracted data were merged into one Excel file organized into categories based on the data extraction tool, see Supplementary File 2. The primary categories that structured the data extraction process are shown in Table 2.

The alternatives for time allocation in guided DMHIs were defined as follows:

1. Direct patient-related tasks online: Synchronous or asynchronous chats and messaging, emails to patients, checking homework, assignments, and test results.
2. Direct patient-related contact offline: Video- or phone calls, face-to-face meetings, diagnostic interviews
3. Indirect patient-related time: Preparation, documentation, referrals, contact with other HCPs, supervision, training.
4. Non-patient-specific professional time: Other DMHI related time: Training, updates, technical support.

**Table 2:** Main categories of data extraction.

Category	Items extracted
1. Context	• Country of origin • Health system type (public, private, mixed) • Setting (academic, established online clinic, routine care in primary care or specialist care, mental health services for specific groups, other)
2. Guided DMHI characteristics	• Level of guidance (number and duration of contacts, stepped care models) • Mode of delivery (web platform, mobile app, text messaging, other) • Target population (specific diagnosis, cross-diagnostic, comorbidities) • Duration of treatment (weeks/modules) • Stepped care of fixed-intensity model • Therapeutic approach
3. Time allocation	• Amount of therapist or other HCP time reported • Categories of time allocation (direct patient contact time, other patient-related time, other DMHI related time) •Time per session/contact (if reported) •Method of time measurement (self-report, log data, estimation, time tracking system)
4. Level of implementation	• Study design (RCT, hybrid effectiveness-implementation, feasibility/pilot, observational routine care study) • Implementation stage (research setting, early implementation, embedded in routine care) • Current scale and reach (single site vs multi-sites vs nation program)

### 2.6 Data analysis and synthesis

Time allocation was analysed for all articles based on the total time reported. When specified, the total time was further broken down into the predefined categories of time allocation: direct patient-related contact online, direct patient-related time offline, and other DMHI-related time. Descriptive statistics were also applied to summarize DMHI duration, type of intervention, health care setting, and number of participating patients. In cases where two online treatment intensities were compared, the reported time of the more intensive alternative was included e.g. (Andrews et al., 2023). If therapist time was estimated and a from-to value reported, the mean time between the two endpoints was included e.g. (Benton et al., 2016). If diagnostic interviews were included in a general admin time, the total time reported was included.

Where available, the following information was extracted: mean and standard deviation (SD) or median and interquartile range (IQR) for all time categories. Using individual means and SDs, an overall mean and SD were calculated. To assess the influence of setting, we compared studies conducted in routine care to those in a research setting, employing *t*-tests and regression analysis. Research studies were defined as feasibility studies, randomized controlled trials (RCTs), or quasi-experimental studies with a control condition. Prospective studies were those reporting routinely collected data, either prospectively or retrospectively. SPSS vs.30 and Microsoft Excel were used for data analysis.

## 3 The case study: time allocation in eTreatment Bergen

### 3.1 Setting

eTreatment in Bergen is part of a national strategy to implement and increase the use of digital mental health interventions (DMHIs) in Norway. It offers online, therapist-supported treatment for common mental health conditions such as anxiety, social anxiety, depression, insomnia, irritable bowel syndrome, adult Attention-Deficit/Hyperactivity Disorder (ADHD), and gaming addiction. Several of the programs are part of routine care, while others are still research projects but embedded within routine clinical services.

Patients may be referred by their general practitioner, specialist health care services, or through self-referral, depending on their presenting problem. If eTreatment is considered suitable, the patient undergoes a diagnostic interview and is then assigned to an appropriate program. They complete treatment modules at their own pace, with a recommended frequency of one module per week. Therapists provide asynchronous support via email at least once a week and monitor patients' assignments and messages.

### 3.2 Methods

Therapists were asked to register their time allocation during a typical week. They used custom-designed registration templates to record the number of minutes spent on each patient every day, divided into two categories:

1. Patient-related tasks carried out on the treatment platform, such as messaging, monitoring progress, and reviewing assignments.
2. Patient-related tasks outside the platform, including diagnostic or interim interviews, end-of-treatment interviews, communication with other healthcare professionals, documentation, and handling referrals.

Moreover, therapists were asked to indicate at which point during the treatment course patients were in order to avoid a concentration of patients at the beginning of treatment and the resulting clustering of diagnostic interviews.

We did not attempt to register other areas of time allocation not related to individual patients, such as general administrative tasks, courses, meetings as these were not regarded as specific to online treatment. Furthermore, time spent on patients who did not enter a treatment program was not reported.

In a second round, therapists were asked to fill a more detailed template for only 3 h on a typical day. This template contained more detailed categories of indirect patient-related time allocation as described in 2 above.

Data were compiled and presented descriptively as means and standard deviations. The Regional Ethics Committee confirmed that there was no need for ethical approval, registration number 847738.

## 4 Results

### 4.1 Literature review: characteristics of the studies included

**Fig. 1:**
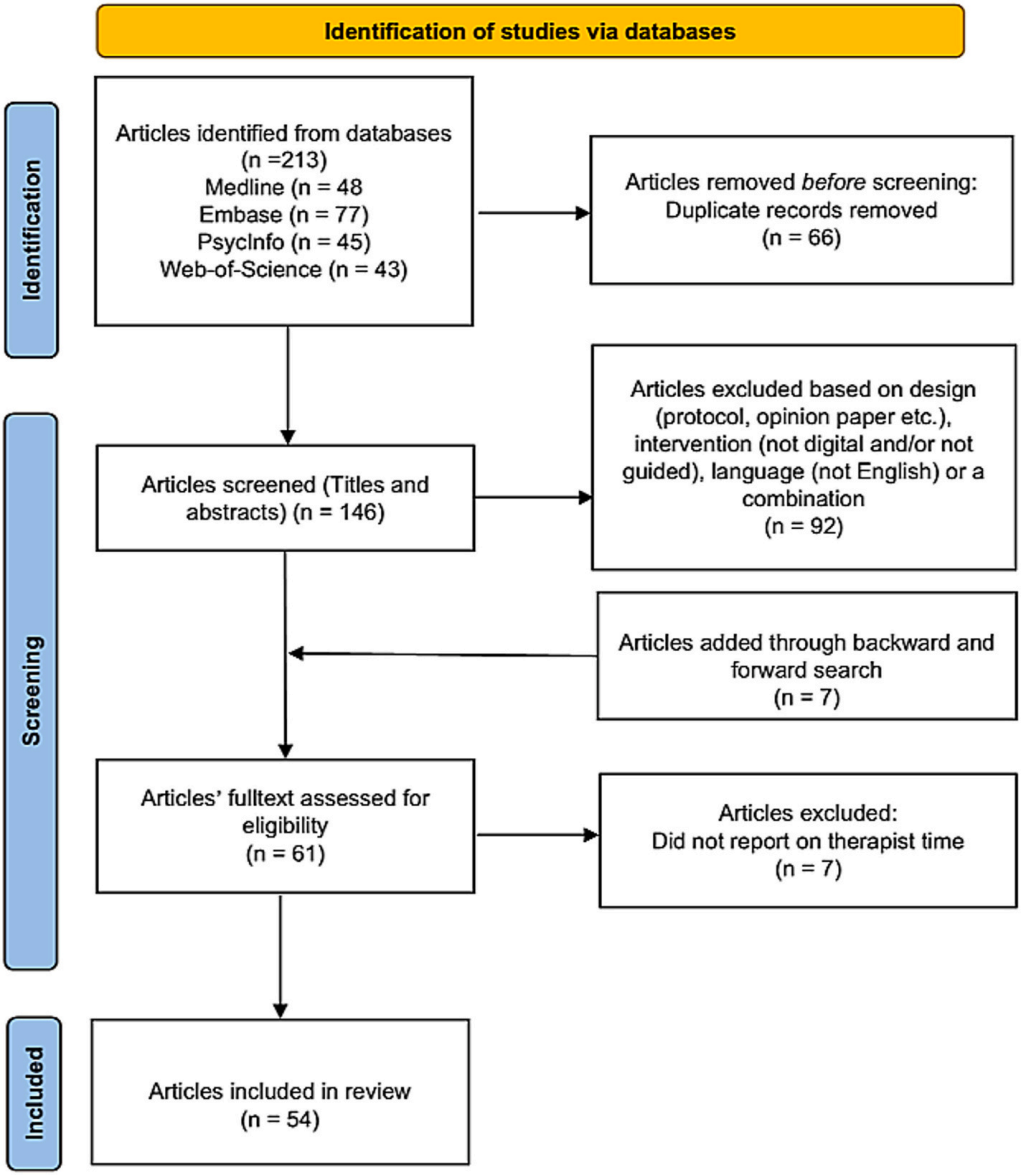
Flowchart.

The first search strings yielded combined 116 citations on 20 May 2025, another updated search on 20 February 2026 yielded 46 more citations. A first screening excluded 92 articles based on criteria that could be inferred from title and abstracts, such as protocol article, opinion paper or wrong type of intervention. All other were retained for full text screening with a focus on time allocation reported. This information could frequently not be inferred from title and abstract alone. Backward and forward search added another 7 citations resulting in 60 articles included in the full text screening, see PRISMA extension for Scoping Reviews flowchart, Fig. 1.

In total, 54 studies were included in the review with the earliest dating from 2005 (Wright et al., 2005) and the most recent from 2026 (Knapstad and Smith, 2026; Karlsson-Good et al., 2026), were included. There was an overweight of Australian studies which contributed with 25 of 54 studies (46.3%), followed by 13 studies (24.1%) from Sweden and five (9.3%) studies from the US. The remainder originated from Canada, Finland, the Netherlands, the UK, Norway, and Singapore.

Most guided DMHIs targeted the most frequent psychological disorders, depression (15%), e.g. (Warmerdam et al., 2010; Kooistra et al., 2019) social anxiety (15%), e.g. (El Alaoui et al., 2017; Hedman et al., 2011) and panic disorder/general anxiety 17%), e.g. (Johnston et al., 2011; March et al., 2023). A few addressed other psychiatric conditions, such as Obsessive/compulsive disorder (OCD) (Wootton et al., 2021; Kwek et al., 2024), Body Dysmorphic Disorder (BDD) (Flygare et al., 2023), and several offered treatment for the psychological aspects of pain and chronic conditions (Dear et al., 2023; Dear et al., 2025).

The dominant therapeutic approach was cognitive behavioural therapy (CBT), utilized in all studies either as the only approach mentioned or in combination with other strategies such as emotion regulation (Andrews et al., 2023) or as exposure and response prevention (Feusner et al., 2022), or condensed prolonged exposure (Bragesjö et al., 2025). According to our definition, 12 studies were conducted in routine care, the majority of 42 were research studies. The number of patients included was larger in routine care, a total of 26,879 patients, mean 1241.00, SD 2873.44, range 225–10,393, than in research studies, total 6519, mean 155.21, SD 153.13, range 17–676.

### 4.2 Duration of treatment

**Fig. 2:**
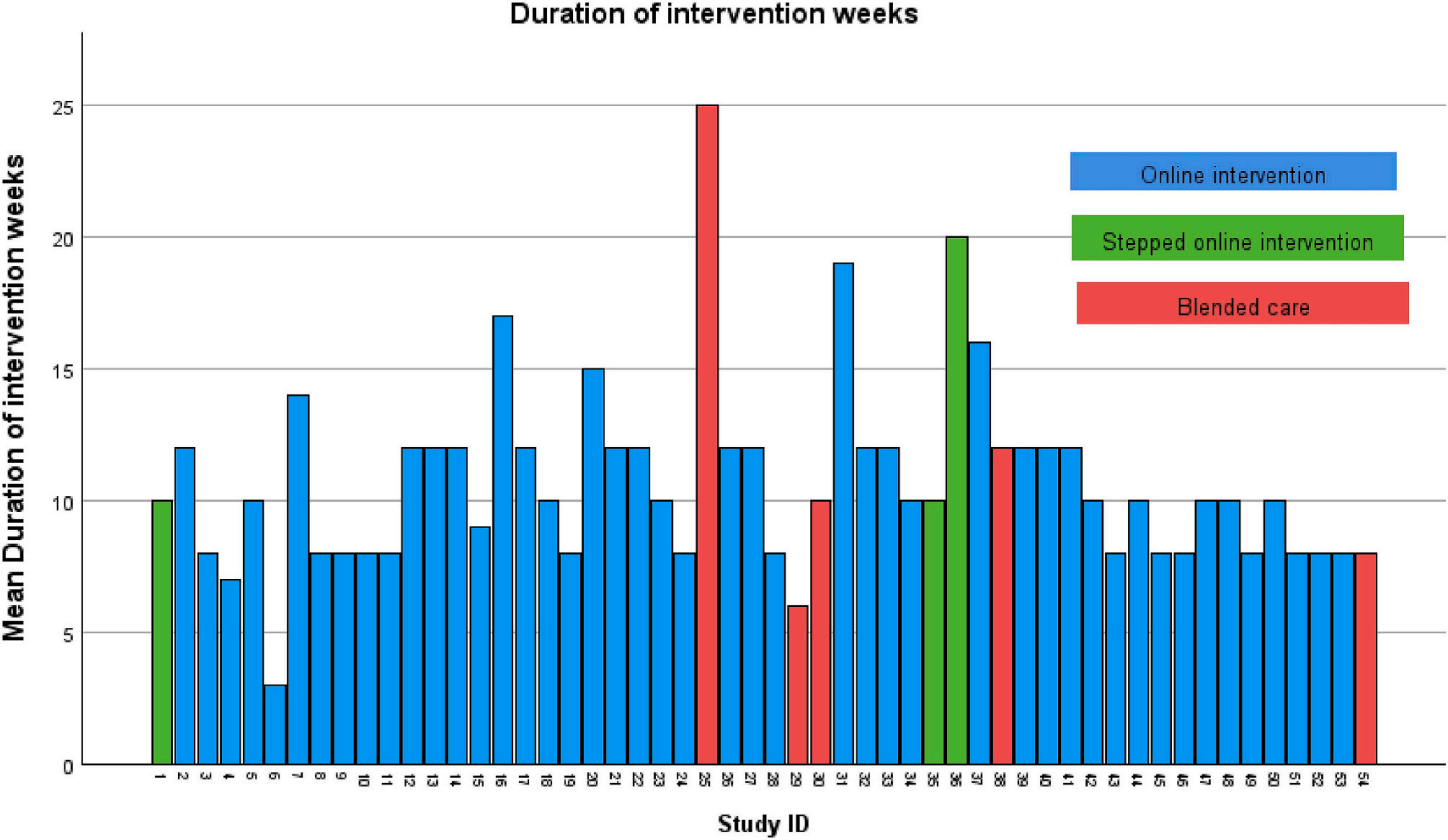
Duration of interventions.

The mean duration was 10.6 weeks (SD 3.6), (range 3–25), median 10 weeks (IQR 4). The most frequent durations of the treatment courses were 8–12 weeks with 16 studies (28.3%) lasting for 8 weeks, e.g. (Aydos et al., 2009; Dear et al., 2022), another 15 studies (27.8%) for 12 weeks e.g. (Kiropoulos et al., 2008; Lindsäter et al., 2019), followed by 12 studies (22.2%) lasting for 10 weeks, e.g. (Kwek et al., 2024; Ljótsson et al., 2010), see Fig. 2 for a graphical depiction.

In most treatment programs patients were supposed to work with roughly one module per week. The shortest duration was 3 weeks with 4 modules (Bragesjö et al., 2024). Several studies provided booster sessions after completed treatment, e.g. (Spence et al., 2011; Andrews, 2010; Clark et al., 2023b). It was difficult to use the number of modules or lessons or phone sessions as a measure of dose, since they varied in length and content. Most importantly, patients did not complete all treatment modules or make use of all available coaching sessions. For example, Hedman et al. reported that participants completed an average of 7.16 modules, while Dear et al. found that only 69% of participants completed all five modules (Dear et al., 2022; Hedman et al., 2013).

### 4.3 Time allocation

Therapists or other HCPs spent a mean 214.57 min (3 h 35 min), SD 214.77, range 46–1198 min, median 146.60 min, IQR 142.98 min, per patient per intervention considering patient-related work on the platform plus phone contact plus one or more diagnostic interviews in studies where this was reported. For blended interventions, face-to-face contact was included, see Table 3 for an overview of studies. It was not possible to estimate the time spent outside the treatment platform other than the diagnostic interviews in online interventions, and face-to-face contact in blended interventions, since only few studies (Axelsson et al., 2020; March et al., 2023; Hedman et al., 2013; Kenter et al., 2015) quantified and/or estimated other patient-related, administrative time, or training time, see Supplementary File 3.

Time allocation was examined with regression analyses, using time allocation as the dependent variable and duration of intervention, diagnosis, and type of intervention (online, stepped online, and blended care) as independent variables. The regression analysis revealed some association between the duration of an intervention and the time allocated by therapists. Duration of intervention explained 41% (adjusted R square 0.407) f the number of minutes spent per treatment, suggesting a substantial effect.

**Table 3:** Overview of studies.

Authors/year	Target group/sample size	Setting^a^/country	Intervention duration and design	Time minutes^b^
Andrews et al. 2023	Transdiagnostic/103	University/community mental health service - Australia	Life Flex program with 8 modules (intro +6 core + booster), two levels of support low-intensity (10 min per week) support, high-intensity (50 min per week)	412
Axelsson et al. 2020	Health anxiety/204	Primary care clinics - Sweden	ICBT: 12 modules over 12 weeks with therapist feedback after each module, outreach calls if inactive.	120
Aydos et al. 2009	Social phobia/17	Outpatient care - Australia	6 online lessons within 8 weeks, homework summaries, secure peer forum, regular clinician emails, telephone contact offered.	180
**Benton et al. 2016**	**Anxiety/1241**	**University counselling center - USA**	**7-week program, 7 modules, weekly 10–12** **min video conference (≈7 contacts), personalized text messages ∼3 per week**	**77**
Bragesjö et al. 2025	PTSD/30	Online platform - Sweden	3-week program, therapist guidance across 4 modules, reminders if inactive ≥3 days, controlled follow-up at week 7	46
Bragesjö et al. 2024	PTSD/102	Public outpatient care – Sweden	10-week program, 6 modules, therapist guidance via platform and phone	130
Clark et al. 2022	Social anxiety/102	Public services - UK	14 weeks treatment, 8 core modules + optional modules, weekly phone or video calls, secure messaging.	468
Dear et al. 2018	Pain in spinal cord injury/68	eCentreClinic - Australia	8-week program, 5 online lessons + homework tasks, weekly clinician contact	93.16
Dear et al. 2021	Chronic pain/490	eCentreClinic Australia	8-week program, 5 online lessons, weekly 10–15 min calls.	67.7
Dear et al. 2022	Chronic conditions/676	eCentreClinic Australia	8-week program, 5 online lessons + homework tasks, weekly clinician contact	56.7
**Dear et al. 2024**	**Pain/557**	**eCentreClinic, multidisciplinary pain service - Australia**	**8-week program, 5 online lessons, structured timeline, automated emails, clinician support as needed**	**52.3**
Ehlers et al. 2023	PTSD/217	Public NHS - UK	Weekly phase: 12 weeks with online modules + therapist support (messages + short weekly phone calls, ∼20 min each), Booster phase: 3 monthly phone calls over next 3 months	300
**El Alaoui et al. 2015**	**Social anxiety/764**	**Outpatient psychiatric clinic - Sweden**	**12 modules, homework, online guidance**	**151.2**
**El Alaoui et al. 2015**	**Social anxiety/654**	**Outpatient psychiatric clinic - Sweden**	**ICBT, Standard duration initially 15 weeks, shortened to 12 weeks, stepwise module access at ∼1 module/week pace, therapist responses within ∼36 h on workdays.**	**139.8**
**Feusner et al. 2022**	**OCD/3552**	**Telehealth outpatient setting - US**	**Twice-weekly 60-min ERP sessions for 3 weeks, then weekly 30-**min **check-ins for 6 weeks, asynchronous messaging support**	**636**
**Feusner et al. 2025**	**OCD in children/2173**	**Telehealth outpatient setting US**	**Weekly or twice-weekly 60-min ERP sessions for 3 weeks, Continuation: 10–14 weeks of weekly 60-min sessions, some transitioned to 30-min check-ins, Parent/caregiver involvement, asynchronous messaging, app-based ERP tools**	**690**
Flygare et al. 2023	BDD/94	Specialized academic medical centre - Sweden	BDD-NET: 8 text-based modules over 12 weeks with worksheets/homework, asynchronous therapist support via built-in email (responses ≤36 h on weekdays).	188.4
Gandy et al. 2023	Neurological disorders/221	Public eCentre Clinic - Australia	Wellbeing Neuro Course: 6 online lessons over 10 weeks, clinician support via weekly phone calls and secure messages.	95.7
Guliani et al. 2022	Transdiagnostic/675	Government funded ICBT clinic - Canada	The Wellbeing Course, 8-week transdiagnostic ICBT, five CBT lessons, email/phone support.	155
Hedman et al. 2011	Social anxiety/126	Public specialist health care, online clinic - Sweden	ICBT: 15-week program with 15 text modules (self-help + homework), therapist feedback each week via secure messaging, no face-to-face/phone.	82.5
**Hedman et al. 2013**	**Panic disorder/570**	**Public specialist health care, online clinic - Sweden**	**Guided Internet-based CBT based on Clark et al., 10-module self-help program, optional anonymous patient forum, therapist feedback after each module.**	**119**
**Hedman 2014**	**Depression/1203**	**Public specialist health care, online clinic – Sweden**	**10 text modules with homework, optional anonymous forum, therapist feedback gated by module completion.**	**148.8**
Johnston et al. 2011	Anxiety disorders/124	Research setting - Australia	8 online lessons over 10 weeks, weekly contact via phone/email, follow-up at 3 months	69
**Kenter et al. 2015**	**Anxiety and depression/4448**	**Routine care -Netherlands**	**Blended care, 4–5 online modules, + face-to-face on average 24.5 sessions**	**1198**
Karlsson-Good et al. 2026	Anxiety and depression/124	Public health system – Sweden	8-week program, 3 transdiagnostic weeks +4 tailored weeks + relapse prevention	66
Khan et al. 2021	Tics/112	Research in NHS – UK	10 chapters over 10–12 weeks, therapist-supported, messaging - phone	143
Kiropoulos et al. 2008	Panic disorder, agoraphobia/86	Primary care/university research clinic - Australia	12-week program, ICBT: Intro +4 learning modules + relapse prevention.	352
Klein et al. 2010	Panic disorder/57	Outpatient/research clinic - Australia	8-week program, ICBT: Intro +4 modules + relapse prevention, therapist contact varied by group	308.3
Knapstad et al. 2026	Anxiety and depression/390	Primary care - Norway	6-module guided iCBT, weekly support sessions.	144.4
Kooistra et al. 2019	Depression/102	Outpatient specialized mental health care centers	Blended CBT protocol: 10 face-to-face sessions +9 web-based sessions with ∼10-week schedule (1 face-to-face +1 online +1 therapist feedback per week).	840
Kwek et al. 2024	OCD/25	Public mental health Services - Singapore	10-week program, 10 modules, therapist support via email and optional phone calls	129
Lenhard et al. 2014	OCD/21	Outpatient mental health service - Sweden	12-week program, 12 chapters for adolescents +5 parent chapters, therapist support via messages and phone calls	233.8
Lindsater et al. 2019	Stress-related disorders/100	Public health care - Sweden	12-week program with 12 text-based modules, weekly written feedback, average modules completed 9.2 (SD 3.2).	87
Ljøtson et al. 2010	Irritable Bowel Syndrome/85	Public health care - Sweden	10-week text-based self-help manual delivered in five sequential steps. Homework reporting required to unlock next step, encouraged to write ≥1 message/week to therapist	165
March et al. 2023	Child and adolescent anxiety/137	Community/online	10 weeks, first 5 sessions self-guided, mid treatment assessment, non-responders stepped up to therapist email for sessions 6–10 + 30 min phone call, responders continued self-guided	180
Mohr et al. 2020	Depression/312	Academic setting - USA	Up to 20 weeks, iCBT: 4 lessons/week, initial 30–40 min call, weekly 10–15 min calls +2–3 messages/week.	315.6
Murphy et al. 2020	Cancer with comorbid anxiety and depression/114	Oncology outpatient setting - Australia	8 lessons over 16 weeks, structured modules, weekly therapist support with email and phone contact.	64.3
Nakao et al. 2018	Major depression/40	Hospital-based outpatient psychiatric care - Japan	12 weeks total. Therapist contact: 12 × 45 min face-to-face sessions (up to 540 min total), web modules: 5 core components. Medication visits roughly every 2 weeks.	540
Perini et al. 2009	Depression/45	Academic setting - Australia	6 online lessons over 8 weeks, homework, weekly email, moderated forum	141
Rapee et al. 2021	Anxiety, children and adolescents/281	University Clinic - Australia	Low-intensity: self-help materials + up to 4 phone sessions (30–40 min each).	111.3
**Ritola et al. 2022**	**Generalized Anxiety Disorder/1099**	**Centrally delivered, referrals from primary care – Finland**	**ICBT, 12 consecutive sessions + 3-month follow-up, recommended 1 session/week, enforced ≥24** **h between sessions, email prompts and login reminders, therapist asynchronous feedback via written messages ≥4 times per therapy**	**132**
Robinson et al. 2010	Generalized Anxiety Disorder/150	Virtual clinic - Australia	6 online lessons over 10 weeks, weekly homework, weekly supportive contact, clinician-assisted group had moderated forum	81
Schniering et al. 2022	Comorbid anxiety and depression, adolescents/91	University clinic - Australia	8 online modules over 8 weeks, supported by 8 × 30-min therapist phone consultations (4 sessions included caregiver)	240
Spence et al. 2011	Anxiety, adolescents/115	University clinic - Australia	10 weekly CBT sessions for adolescents +5 parent sessions, BRAVE-ONLINE program with interactive modules, quizzes, homework, minimal therapist support (email + one phone call), booster sessions at 1- and 3-months post-treatment	150
**Titov et al 2015**	**Anxiety and depression/10,393**	**Nationwide online clinic Australia**	**Four structured courses: Wellbeing, Wellbeing Plus, OCD, PTSD, 4–6 lessons over 8 weeks, homework assignments, automated emails, weekly therapist contact**	**171.8**
Titov et al. 2008	Social phobia/105	Public/Research setting - Australia	6 online lessons over 8 weeks, homework, secure forum, regular email contact with therapist.	150
Titov et al. 2008	Social phobia/88	Public/Research setting - Australia	6 online lessons over ∼10 weeks, homework, secure forum, regular therapist email contact (∼22 emails) + forum responses.	151,8
Titov et al. 2008	Social phobia/98	Public/Research setting - Australia	6 online lessons over ≤10 weeks, homework, online forum. Therapist emails after each lesson, weekly reminders, forum moderation, phone outreach if inactive.	193
Titov et al. 2010	Depression/141	Public/Research setting -Australia	6 online lessons over 8 weeks, weekly homework, weekly supportive contact (email or phone), clinician group had moderated forum	60.5
Titov et al. 2011	Transdiagnostic/77	University-affiliated online clinic - Australia	8 online lessons over 10 weeks, weekly clinician contact, access to moderated discussion forum, automated emails. Weekly telephone calls and secure instant messaging/email, automated reminders/emails.	124.8
Warmerdam et al. 2010	Depression/263	Research setting - Netherlands	ICBT (8 weekly lessons + booster at week 12) Internet-based problem-solving therapy (5 weekly lessons).	160
Wims et al. 2010	Panic disorder, agoraphobia/59	Online clinic - Australia	6 online lessons over 8 weeks, homework, moderated forum, weekly email contact	135
**Wootton et al. 2021**	**OCD/225**	**National online platform - Australia**	**Initially 6 lessons over 8 weeks, subsequently 5 lessons over 8 weeks, delivered self-guided or clinician-guided, guided support via telephone or secure messaging.**	**90**
Wright et al. 2005	Depression/45	Outpatient psychiatric clinic affiliated with University - USA	Face-to-face blended with computer program including videos and interactive exercises, 9 sessions over 8 weeks. First session 50 min, subsequent sessions 25 min + 20–30 min computer work immediately after each session.	250

Abbreviations: BDD: Body Dysmorphic Disorder, CBT: Cognitive Behavioural therapy, ERP: Exposure and response prevention, FTF: face-to-face treatment, OCD: Obsessive Compulsive Disorder.

a Routine care studies are in bold.

b Admin times were included if they comprised diagnostic interviews and assessments. Admin times without patient contact were disregarded as only reported in few studies.

There were differences with regard to diagnosis. Depression and OCD required on average most therapist time. Time spent in depression interventions was mean 346.23 min, SD 333.32, in OCD mean 355.80 min, SD (285.97. However, with large variation and not reaching statistical significance.

The type of intervention, blended, or online, or stepped online care, or did not influence how much time therapists spent, adjusted R square 0.01. However, only few blended (*n* = 5) or stepped-care studies (*n* = 3) were included (Wright et al., 2005; Andrews et al., 2023; Knapstad and Smith, 2026; Kooistra et al., 2019; March et al., 2023; Kenter et al., 2016; Mohr et al., 2019; Nakao et al., 2018). Time allocation was not affected by the setting, research study vs. routine care, *p* = 0.134.

### 4.4 Categories of time allocation

Predominantly, only direct patient-related work was reported, including phone calls, video meetings, messaging, diagnostic interviews, monitoring homework and assignments related to an individual patient within the online platform or in combination with face-to-face sessions in blended approaches. It was not always clear if therapist time was measured or estimated. Frequently therapist time was derived from logs or records or measured in another way, e.g. in all studies by Titov et al. (Titov et al., 2015; Titov et al., 2011; Titov et al., 2010; Titov et al., 2008a; Titov et al., 2008b; Titov et al., 2008c). Where a diagnostic interview was mentioned and quantified, it was added to therapist time, including follow-up interviews, as e.g. in Andrews et al. (Andrews et al., 2023) and Aydos et al. (Aydos et al., 2009). Other not directly patient-related categories included training, supervision and administrative tasks. These were not detailed in most studies, however, estimated in some, as in Hedman et al. reporting <5 h per patient, and Robinson et al. 7 h per week for all patients (Hedman et al., 2013; Robinson et al., 2010). For an overview of categories of time allocation mentioned see Table 4.

**Table 4:** Categories of time allocation.

Time category	Task	Relationship to digital delivery
Direct patient contact	Diagnostic and eligibility assessment	Digitally mediated, but not digital-specific. Assessment is common across modalities and may be conducted by video, telephone, digitally administered measures, or in person.
	Video session	Digitally mediated. A conventional synchronous clinical encounter conducted through video.
	Telephone call	Digitally mediated, but not necessarily specific to DMHIs. Telephone contact also occurs in FTF care.
	Synchronous text/chat session	Digital-specific mode of contact.
	Asynchronous messages, texts, or emails	Characteristic of guided DMHIs. Common forms of human support include supportive messages, personalized feedback, reminders, and responses to patients' questions.
	Follow-up and engagement contacts	Potentially modified. Follow-up is common across modalities, but digital interventions may use automated or personalized reminders, messages, or telephone/video calls.
	Direct crisis or risk-management contact	Digitally mediated, but not digital-specific. Risk management is a general clinical responsibility, although remote care can require additional procedures for confirming location, contacting emergency services.
Indirect patient-related time	Monitoring symptoms, progress, engagement, or adherence	Potentially modified and sometimes platform-enabled. Digital systems can provide symptom scores, activity data, completion records, and alerts, however, clinician review still consumes time unless it is fully automated.
	Reviewing homework or platform submissions	Characteristic of guided DMHIs, but not unique to them. Homework review is also part of FTF CBT, digital platforms change how work is submitted, accessed, and reviewed.
	Preparing personalized feedback	Characteristic of guided DMHIs. Written feedback based on completed modules or exercises is a commonly reported guidance activity in DMHI.
	General preparation and care planning	Not inherently digital-specific but potentially modified. Platform data may reduce or increase preparation time depending on usability and workflow integration.
	Reviewing risk alerts or questionnaire responses	Potentially platform enabled. Digital measures may generate automated flags, but professional review and decisions remain patient-related clinical work.
	Documentation and medical records	Not inherently digital-specific but potentially modified. Time may be affected by interoperability, automatic data transfer, or duplicate documentation across the intervention platform and health record.
	Care coordination and referrals	Not inherently digital-specific. Digital delivery may alter communication channels but does not eliminate this work.
Patient-level technical and administrative time	Patient onboarding and account setup	Digital-specific. May include registration, platform orientation, consent procedures, and help with initial access.
	Patient-specific technical support	Digital-specific. Includes troubleshooting login, device, connectivity, notification, or platform-use problems.
	Scheduling and routine administration	Not inherently digital-specific but potentially modified. Some processes may be automated, while others may introduce additional platform-related work.
Program-level implementation and maintenance	Training for digital provision	Digital-specific implementation activity. Training may cover online communication, remote assessment and risk protocols, platform-supported guidance, privacy, and digital clinical competencies. It should not automatically be treated as per-patient time.
	Technical training and system updates	Digital-specific implementation or maintenance activity. Usually a fixed or recurring program-level resource rather than directly attributable to one patient.
	Supervision and quality assurance	Not inherently digital-specific but may require adaptation. Digital services may require supervision of written feedback, online boundaries, platform use, and remote risk management.
	Workflow development and platform integration	Digital-specific implementation activity. Includes incorporating the intervention into referral pathways, clinical records, staff roles, and existing service routines.
	Platform governance and maintenance	Digital-specific program-level activity. Includes privacy, security, licensing, vendor coordination, updates, and system monitoring.

### 4.5 The case study – eTreatment Bergen

**Fig. 3:**
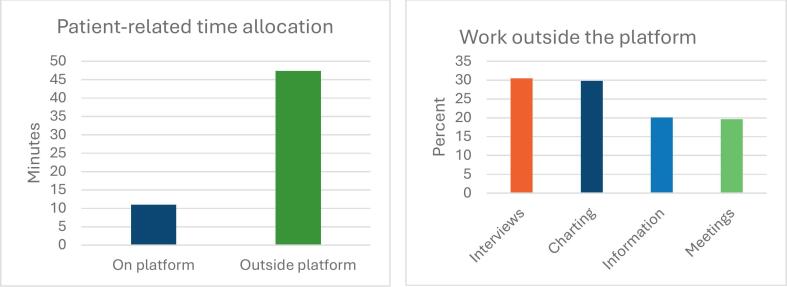
Time allocation - Case study. Interviews comprise diagnostic interviews throughout the treatement course, Charting refers to documentation in the medical records, Information is any communication with colleagues and external health professionals, Meetings comprise patient eligibility review meetings and other staff meetings.

In the case study, 9 therapists registered their patient-related time allocation inside and outside the treatment platform for 95 patients during a typical week. On average, the therapists registered time allocation for 10.55 (SD 5.59) patients. Patients participated in programs for depression (18.9%), anxiety/panic disorder (14.7%) social anxiety (12.6%), ADHD for adults (29.5%), insomnia (4.2%), and mixed/unclear (18.9%). In total, therapists spent 54.95 (SD 26.19), range 11.54–102.50 min per patient per week with individual patient-related work. Based on the regular program course of 12 weeks, this would roughly equate 659.4 min (almost 11 h) per patient per treatment. Work on the treatment platform such as messaging and monitoring activity and homework accounted for 14.56 min (26.50%), SD range 7.5–40.00, and patient-related work outside the platform such as diagnostic interviews, phone calls, documentation, referrals, and assessments accounted for 40.49 min (73.69%), SD 29.04, range 2.00–95.00, see Fig. 3, panel A. The second round of registration focused on the tasks outside the treatment platform. Here, therapists were asked to specify these tasks. Six therapists recorded their time allocation outside the platform on a typical day for 3 h, Fig. 3, panel B. They reported spending almost a third (30.5%) of their time outside the platform on diagnostic interviews which are conducted at the start, midway, and end of treatment. Another third (29.8%) was spent on keeping medical records, while information seeking and exchange and meetings accounted for 20.1% and 19.6% respectively.

## 5 Discussion

With this scoping review and case study, we aimed to estimate therapist time allocation in guided DMHIs to support implementation planning. We focused on 1. the total amount of therapist or HCP time reported and 2. the types of activities or time categories documented. On average, therapists spent 3 h and 35 min per patient per intervention in guided online, stepped care and blended approaches. However, this estimate showed substantial variability, reflected in the large standard deviation and wide range. Moreover, findings from the literature suggest that only a fraction of actual therapist time allocation in guided DMHIs is reported. Most published figures reflected patient-related work on the treatment platform (monitoring progress and reviewing), and direct contact (e.g., messaging, phone and video calls, diagnostic interviews), and did not quantify additional patient-related, treatment-related, or system-related activities, such as charting, training and supervision. This gap may hinder realistic estimates of the time required for implementation and sustainment of guided DMHIs. Incomplete workload/time reporting limits workforce planning, cost modelling, scalability, and sustainability of digital mental health services.

Our pilot case study confirmed this pattern, demonstrating that a substantial proportion of therapist time is spent on tasks conducted outside the platform. When these activities are included, as in our study, the total therapist time increases considerably. This may partly reflect the fact that certain off-platform tasks, such as diagnostic interviews, are typically time-intensive. However, the observation that most therapist time (74%) in our sample occurred outside the platform should be interpreted with caution, as it may not be representative due to the small sample size and the specific characteristics of the context.

We identified several relevant categories of time allocation outside the platform, such as training, supervision, and administrative work. However, in most papers these categories were presented as a single undifferentiated lump sum or not quantified at all e.g., (Johnston et al., 2011; Lindsäter et al., 2019; Hedman et al., 2013). This is understandable given that documenting detailed time allocation can be tedious, and time logs may itself influence the results and change behaviour (Chatzitheochari et al., 2018). It is important to acknowledge that activities outside direct patient contact are inherent to all therapy modalities and are not unique to DMHIs. Nevertheless, including time devoted to documentation, training, supervision, and related tasks in reported time allocation estimates would provide a more complete understanding of the overall distribution of clinical workload. A substantial underestimation of the time allocation needed when delivering guided DMHIs may raise unrealistic expectations and cause frustration when these are not fulfilled.

### 5.1 How much time is actually reported and is there a large variation?

The mean time spent during a guided treatment course was 214.57 min or 3 h 35 min. This is in almost all cases substantially less than equivalent face-to-face therapy would require, and consequently more cost-effective across diagnostic groups. For comparison, standard individual face-to-face CBT typically involves 10–15 sessions of 45–60 min each, corresponding to approximately 450–900 min (7.5–15 h) of direct therapist contact. For instance, among the studies that had an active face-to-face comparison in this review, Clark et al. reported 4.1 h for the online intervention and 18.4 h for face-to-face treatment (Clark et al., 2023a), and Axelsson reported 78% lower therapist time (Axelsson et al., 2020). Unsurprisingly, treatment duration did influence time reported to a substantial degree.

This cost-effectiveness has been confirmed, among others, in studies by El Alaoui et al. for social anxiety, Khan et al. for social anxiety, panic disorder and depression, and Bragesjö et al. for trauma (El Alaoui et al., 2017; Bragesjö et al., 2025; Khan et al., 2025). Yet, as pointed out by Khan et al., there may large variations due to the patient volume in relation to the resources needed to set up the online treatment (Khan et al., 2025). In the present review, we also found a large variation in minutes of therapist time spent during a treatment course, ranging from 46 (Bragesjö et al., 2025) to 1198 min (Kenter et al., 2015). Notably, even the upper end of this range overlaps with the lower boundary of typical face-to-face CBT time requirements, while the majority of guided DMHI interventions remain well below standard face-to-face workload levels.

### 5.2 Contributors to therapist time

Treatment duration played a role in how much time the therapist spent and was the only significant contributor, as opposed to diagnosis and type of treatment. However, it is not the only factor to explain the large variation even within one patient group. Examples of this variation are social anxiety, where Clark et al. (Clark et al., 2023a) reported 468 min and Titov et al. (Titov et al., 2008a) 193 min, or depression with 149 min reported by Hedman et al. (Hedman et al., 2013) and 60.5 by Titov et al. (Titov et al., 2011). These figures suggest that differences in intervention structure and therapist contact intensity, rather than diagnosis per se, may account for variability in workload, whereas face-to-face CBT typically follows a more standardized session-based format with less flexibility in contact time. It remains unclear if the duration of a treatment course is based on clinical experience and/or derived from face-to-face CBT, the severity of symptoms, or pragmatic considerations concerning the resources available. The latter is especially relevant in routine care. In many cases, the weekly module structure of guided DMHIs mirrors traditional CBT session frequency. Yet, it replaces fixed 50-minute sessions with shorter asynchronous contacts distributed across the week, which likely contributes to the overall reduction in direct therapist time and adds flexibility. In this context the study by Kenter et al. is an interesting outlier as they found an increase of therapist time in a blended ICBT intervention compared to face-to-face treatment (Kenter et al., 2015). The authors attributed the increased therapist time to a lack of clear guidance during implementation which led therapists to treat the online component as an add-on rather than an integrated part of therapy. Another study by Kooistra et al. found roughly equal therapist time allocation in a blended face-to-face and online approach compared to standard face-to-face treatment (Kooistra et al., 2019). These findings illustrate that time savings are not inherent to digital delivery per se but depend on appropriate integration into clinical workflows. When online components are layered onto existing face-to-face treatment rather than substituting parts of it, overall therapist workload may increase rather than decrease. This emphasizes the need for training and guidelines for therapists and other HCPs when providing online treatment.

In our case study, therapists spent almost 3/4 of patient-related time outside the treatment platform. It is difficult to tell, if these results are representative, given the lack of information in the literature. To complicate things further, many tasks are part of any therapy and thus not specific to guided DMHIs, for instance supervision and patient-related communication with other HCPs. Time logs of these will only provide information about general time allocation in psychological treatments. Therapists were instructed to report only patient-related time, and examples were provided in the checklist to support consistent reporting. However, certain activities may be difficult to classify. For instance, a supervision session focused on a particular patient may gradually develop into a broader discussion. In addition, the interviews conducted at treatment initiation, midway through treatment, and at termination may themselves represent a considerable time investment, while written communication can be a particularly efficient support modality for experienced online therapists.

### 5.3 Can online therapy and time allocation become more personalized?

Independent of face-to-face or online treatment, there will always be some variation on how good patients respond to an intervention, even in the same diagnosis and severity category. This variation is addressed in the stepped-care studies included in this review. Andrews et al. provided optional therapist support, March et al. and Mohr et al. increased support for non-responders (Andrews et al., 2023; March et al., 2023; Mohr et al., 2019). Such stepped-care approaches may further differentiate guided DMHIs from traditional face-to-face CBT, where session length and frequency are typically fixed in advance, and may allow more efficient allocation of therapist time by reserving higher-intensity support for patients who need it most. On the other hand, minimal therapist support, such as a brief onboarding interview and a concluding interview only, may be sufficient for many patients with mild to moderate symptoms. Studies using factorial designs and long-term follow-up, such as the study by Bur et al. (Bur et al., 2022) can help to compare the influence of different levels of support. In the nearer future, promising technologies such as machine learning and natural language processing may help to personalize mental health and adapt their intensity and duration as needed by the patient (Löchner et al., 2025).

### 5.4 Integration into workflows and usability

From an implementation science perspective, an important source of DMHI-specific workload relates to workflow integration, platform usability, and interoperability with existing health information systems (Titov et al., 2019). Research in digital health consistently shows that when platforms are not integrated into routine clinical workflows, they may introduce “hidden work” through parallel documentation, duplicate data entry, and manual coordination across systems (Schueller and Torous, 2020; Zhao et al., 2023; Graham et al., 2020b). If DMHI platforms are not interoperable with electronic health records (EHRs), referral systems, or scheduling software, therapists may need to switch between interfaces and re-enter patient information, increasing both administrative time and cognitive load. Usability research further indicates that interface design strongly influences clinician efficiency (Støme et al., 2021). Socio-technical models of implementation emphasise that digital technologies must align with existing clinical routines rather than requiring clinicians to reorganise their workflow around the platform (Støme et al., 2021). A study on the implementation of guided DMHIs in five countries by Folker et al. identified the referral process as a source of time waste because the programs were not suitable for a substantial fraction of the patients (Folker et al., 2018). According to managers' feedback more selective referral process would contribute to a more efficient time allocation. In the study by Folker et al., and in line with research by Schueller et al. and Strudwick et al., (Schueller and Torous, 2020; Strudwick et al., 2025), user-friendliness, or rather a lack thereof, was described as a potential time thief.

To implement guided DMHIs the most relevant information on time allocation would concern specifics to the mode of delivery including training to familiarise with the platform, maintenance, updates. HCPs should be included in both development and implementation of DMHIs and thus promote acceptability and long-term sustainability (Vis et al., 2018; Greenhalgh et al., 2016).

### 5.5 Learning to provide therapy in a different way requires time

Beyond practicalities differences in the way to convey therapy have to be considered. Bortveit et al. explored therapists' experiences with ICBT and found that they expressed profound differences compared to providing traditional face-to-face therapy (Bortveit et al., 2023). These included the skills needed for written communication, which provided a means to establish contact but at the same time allowed to keep some professional distance. Therapists stated that written communication can be time consuming and emphasised the need to allocate sufficient time. In their review, Ramos et al. highlight the time-consuming nature of learning to use new technologies and note that therapists may find it frustrating when this learning process reduces the time available for actual treatment (Ramos et al., 2024).

### 5.6 Therapists' well-being

While saving therapist time is a key driver for offering DMHI, efficacy cannot be increased limitless as this might affect job satisfaction and mental health of the HCPs. Importantly, digital delivery may reduce synchronous contact time but does not eliminate administrative burden and can introduce new forms of technical and cognitive workload. A recent large survey study by Pathman et al. found that behavioural health care providers face high levels of stress, burnout, and emotional exhaustion related to factors, such as a high caseload and administrative burden (Pathman et al., 2025). This impedes retention of personnel and underscores the need for targeted strategies to promote the well-being of mental health care providers (Johnson et al., 2018). Online therapy may offer health care personnel a more flexible workday because support can be given asynchronously, with options for working from home that may be incorporated in strategies to promote the well-being for health care workers and prevent burn-out.

## 6 Limitations

We may have overlooked several other relevant articles since time allocation frequently was presented as a side information and not as the main outcome. However, we are confident that the range of time allocation presented, and the breadth of the studies included is quite representative for other studies.

Time allocation was inconsistently defined and measured across studies. Most studies relied on therapist self-report or retrospective estimates rather than prospective time-tracking or automated log data. This may have introduced recall bias and imprecision and likely contributed to underestimation of indirect or administrative time. The lack of standardized definitions for direct and indirect time further limits comparability across studies. Admittedly, it can be challenging to distinguish between time spent on tasks that are specific to DMHIs and time spent on activities that are common across treatment modalities. Moreover, the allocation of time to different tasks is likely to vary across settings and contexts. More comprehensive reporting of resource requirements, including time spent on system setup, familiarization, training, and other implementation-related activities, would be valuable for improving transparency and facilitating future research and comparisons across studies.

Our analyses were conducted at the study level rather than the patient level. The regression analysis examining the association between duration and therapist time was therefore ecological in nature and unweighted for sample size. The objective was to summarize time allocation across studies rather than to provide a pooled estimate. Equal weighting was considered more appropriate for addressing the descriptive research objectives of this review. Consequently, the findings should not be interpreted as reflecting patient-level relationships, and smaller studies may have contributed disproportionately to variance estimates.

Moreover, cost-effective use of therapist time is obviously only relevant if a similar symptom improvement can be expected. We did not look specifically at the effectiveness of the studies included. Yet, none of the included studies was inferior if an active control was applied. More important, a solid body of research supports the effectiveness of guided DMHIs, see reviews (Hedman-Lagerlöf et al., 2023; Olthuis et al., 2016).

It is debatable whether such a broad range of studies should have been included, from relatively small feasibility trials, such as those by Aydos et al. and Bragesjö et al. (Bragesjö et al., 2025; Aydos et al., 2009) to large-scale routine care studies such as the studies by Titov et al. and Dear et al. (Titov et al., 2017; Dear et al., 2024). It proved difficult to define clear-cut eligibility criteria based on a single type of setting, such as routine care, study design, or the number of participants. In many studies, a new intervention was added to an already established online clinic or performed in routine care e.g. (Dear et al., 2018; Ehlers et al., 2023; Guliani et al., 2022) meaning that the research took place in routine care. Similarly, several of the smaller studies were also conducted in routine care (Bragesjö et al., 2025; Aydos et al., 2009). Consequently, we chose to apply broad inclusion criteria and instead examine the influence of the setting afterward, however, this proved difficult as there were to many grey areas to make a comparison meaningful. Nevertheless, this heterogeneity in study design, patient populations, intervention formats, and implementation maturity likely contributed to the wide variation observed in therapist time. The inclusion of both pilot trials and large-scale services increases ecological validity but reduces internal comparability.

The case study is based one setting and few observations and by no means representative for the entire field of guided DMHIs. Yet, it illustrates that there can be a lot of work not accounted for when reporting time allocation in guided DMHIs.

## 7 Conclusion

The reported time allocation refers primarily to direct patient contact and monitoring, without accounting for additional patient-related or system-related tasks. Therapist time allocation in guided digital mental health interventions (DMHIs) is inconsistently and incompletely reported in the current literature, with most studies focusing solely on direct patient contact and rarely quantifying indirect or system-level tasks. This lack of comprehensive time estimates may pose challenges for implementation, as it limits accurate estimation of workload, cost, and scalability. However, it must be acknowledged that it can be difficult to differentiate between time spent on tasks that are specific to DMHIs, and time spent on activities that are shared across therapeutic modalities. Nevertheless, even when assuming a substantial amount of time dedicated to these other tasks, the total required time remains considerably lower than what would be expected for an equivalent face-to-face intervention. Consequently, broader implementation should be encouraged, while ensuring that both patient and therapist needs are considered. Future research should adopt standardized reporting of therapist workload, distinguishing between direct, indirect, and program-related activities.

## Declaration of Generative AI and AI-assisted technologies in the writing process

During the preparation of this work the authors used you.com and copilot in order to extract information from the literature and to refine language. After using these tools, the authors reviewed and edited the information from each full text as needed and take full responsibility for the content of the published article.

## Declaration of competing interest

The authors declare that they have no known competing financial interests or personal relationships that could have appeared to influence the work reported in this paper.

## Appendix A Supplementary data

Suppl. File 1 Search-documentation.

Suppl. File 2 Data extraction tool.

Suppl. File 3 Time categories_info.
