# Supplementary material for: Therapists' time allocation in guided digital mental health interventions – A scoping review and case study
Source: Internet Interv. 2026 Jul 30;45:100983. doi: 10.1016/j.invent.2026.100983 (PMC13450580; doi:10.1016/j.invent.2026.100983)
Supplement: Supplementary file 1 [file mmc1.docx]

**Ovid MEDLINE(R) and Epub Ahead of Print, In-Process, In-Data-Review & Other Non-Indexed Citations, Daily and Versions <1946 to May 19, 2025>; Search date 20 May 2025**

1	exp Telemedicine/	53096

2	digital mental health intervention*.ti,ab,kf.	502

3	internet based treatment*.ti,ab,kf.	311

4	telemedicine.ti,ab,kf.	30572

5	telehealth.ti,ab,kf.	18884

6	e-health.ti,ab,kf.	4958

7	mhealth.ti,ab,kf.	12320

8	1 or 2 or 3 or 4 or 5 or 6 or 7	83165

9	exp Depression/	170692

10	exp Anxiety Disorders/	96348

11	depression.ti,ab,kf.	485483

12	anxiety.ti,ab,kf.	311440

13	phobia*.ti,ab,kf.	11029

14	stress disorder*.ti,ab,kf.	47070

15	ptsd.ti,ab,kf.	38973

16	9 or 10 or 11 or 12 or 13 or 14 or 15	757563

17	((therapist* or clinician* or psychologist* or register* or keeping or saved or saving) adj2 (time or hour* or minute*)).ti,ab,kf.	14115

18	8 and 16 and 17	43

<https://ovidsp.ovid.com/ovidweb.cgi?T=JS&NEWS=N&PAGE=main&SHAREDSEARCHID=3jQWVER9tC7LRqGC6RGNsdrWL4oYYYGreF2jmBMud68LtejvIBPEsQGStOyNDqAxL>

**DONE=uploaded**

Comments to OVID databases:

- / = search on subject heading
- Exp = search on a subject heading, expanded to include narrower terms
- Ti,ab,kf = search in title, abstract and authors keywords of the article
- Adjn = The adjacency operator (ADJn) retrieves records that contain search terms within a specified number (*n-1*) of words from each other in any order

**Embase (Ovid) <1974 to 2025 May 19>; Search date 20 May 2025**

1	exp telemedicine/	85492

2	digital mental health intervention*.ti,ab,kf.	410

3	internet based treatment*.ti,ab,kf.	437

4	telemedicine.ti,ab,kf.	39572

5	telehealth.ti,ab,kf.	23365

6	e-health.ti,ab,kf.	6540

7	mhealth.ti,ab,kf.	11339

8	1 or 2 or 3 or 4 or 5 or 6 or 7	118054

9	exp depression/	733290

10	exp anxiety disorder/	370023

11	depression.ti,ab,kf.	670557

12	anxiety.ti,ab,kf.	438017

13	phobia*.ti,ab,kf.	15351

14	stress disorder*.ti,ab,kf.	58388

15	ptsd.ti,ab,kf.	51285

16	9 or 10 or 11 or 12 or 13 or 14 or 15	1328039

17	((therapist* or clinician* or psychologist* or register* or keeping or saved or saving) adj2 (time or hour* or minute*)).ti,ab,kf.	20431

18	8 and 16 and 17	64

<https://ovidsp.ovid.com/ovidweb.cgi?T=JS&NEWS=N&PAGE=main&SHAREDSEARCHID=29wbHHoNCRHdXrlNNeNZDM7bizZKquIhMY4tMmenMma8bXsa1uiNjYZ0c4Xrl7ioi>

**DONE**

**APA PsycInfo (Ovid) <1806 to May 2025 Week 2>; Search date 20 May 2025**

1	exp Telemedicine/	17140

2	digital mental health intervention*.ti,ab.	194

3	internet based treatment*.ti,ab.	248

4	telemedicine.ti,ab.	2927

5	telehealth.ti,ab.	4544

6	e-health.ti,ab.	959

7	mhealth.ti,ab.	1613

8	1 or 2 or 3 or 4 or 5 or 6 or 7	20912

9	exp Major Depression/	181183

10	exp Anxiety Disorders/	46210

11	depression.ti,ab.	309298

12	anxiety.ti,ab.	252742

13	phobia*.ti,ab.	13370

14	stress disorder*.ti,ab.	46221

15	ptsd.ti,ab.	43297

16	9 or 10 or 11 or 12 or 13 or 14 or 15	531307

17	((therapist* or clinician* or psychologist* or register* or keeping or saved or saving) adj2 (time or hour* or minute*)).ti,ab.	2793

18	8 and 16 and 17	44

<https://ovidsp.ovid.com/ovidweb.cgi?T=JS&NEWS=N&PAGE=main&SHAREDSEARCHID=78iPAlRs1oOFQPGGwdjXBWsFKjqYxbmCZ904jula0f6EaaHoBkxjouTfWxnukJbCW>

**DONE**

**Web of Science (Clarivate), Web of Science Core Collection, covering WOS.SCI: 1945 to 2025, WOS.AHCI: 1975 to 2025, WOS.ESCI: 2020 to 2025, WOS.SSCI: 1956 to 2025. Search date 20 May 2025**

TS=(((telemedicine or "digital health intervention*" or "internet based treatment" or telehealth or e-health or mhealth) AND (depression or anxiety or phobia* or "stress disorder*" or PTSD) AND ((therapist* or clinician* or psychologist* or register* or keeping or saved or saving) NEAR/1 (time or hour* or minute*))))	41 hits
